# Association between heart rate and cardiovascular death in patients with coronary heart disease: A NHANES‐based cohort study

**DOI:** 10.1002/clc.23818

**Published:** 2022-03-30

**Authors:** Ruicong Ma, Jianbo Gao, Shiyuan Mao, Zhirong Wang

**Affiliations:** ^1^ Department of Cardiology The Affiliated Hospital of Xuzhou Medical University Xuzhou Jiangsu China

**Keywords:** cardiovascular death, coronary heart disease, heart rate, NHANES

## Abstract

**Background:**

Due to the lack of research, this study aimed to assess the association between the specific range of heart rate and cardiovascular (CV) death in coronary heart disease (CHD) patients.

**Hypothesis:**

Heart rate of 70–79 bpm may be associated with reduced risk of CV death in CHD patients.

**Methods:**

This retrospective cohort study collected the data of CHD patients from the eight cycles of the Health and Nutrition Examination Survey (NHANES). The included patients were divided into four groups: <60, 60–69, 70–79, and ≥80 bpm. The start of follow‐up date was the mobile examination center date, the last follow‐up date was December 31, 2015. The average follow‐up time was 81.70 months, and the longest follow‐up time was 200 months. Competing risk models were developed to evaluate the association between heart rate and CV death, with hazard ratios (HRs) and 95% confidence intervals (CIs) calculated.

**Results:**

A total of 1648 patients with CHD were included in this study. CHD patients at heart rate of <60 (HR, 1.35; 95% CI, 1.34–1.36), 60–69 (HR, 1.05; 95% CI, 1.04–1.06) or ≥80 (HR, 1.39; 95% CI, 1.38–1.41) bpm had a higher risk of CV death than those at heart rate of 70–79 bpm.

**Conclusions:**

Heart rate of <70 or ≥80 bpm was associated with an elevated risk of CV death among CHD patients. Continuous monitoring of heart rate may help to screen for health risks and offer early interventions to corresponding patients.

## 1 INTRODUCTION

Coronary heart disease (CHD) is still a major cause of morbidity and mortality around the world, featured by chronic immune‐inflammatory and fibro‐proliferative disease induced by lipids.^1^, ^2^ CHD accounts for 27% of total cardiovascular disease (CVD) costs in Europe,^3^ and this condition leads to around one third of all deaths for people aged over 35 years although the mortality gradually declines in western countries.^4^ In China alone, of the about 290 million CVD patients, 11 million suffer from CHD,^5^ with increasing morbidity and mortality.^6^ The manifestations of CHD compose approximately two thirds of developed cardiovascular (CV) events.^7^ Individuals with CHD are often afflicted with CV events, such as heart failure, stroke, myocardial infarction, and cerebral thrombosis, and even die of these events.^8^, ^9^ Identifying the factors associated with CV death is then of significant necessity for the risk evaluation and management of CHD.

Resting heart rate is central to cardiac output that is easy to measure as a parameter,^10^, ^11^ which can be used to assess CV health and the risk of CV events.^12^, ^13^ Heart rate has been recognized as an independent predictor of CV mortality in the general population and patients with CVD.^14^ Increased resting heart rate is a modifiable risk factor for CV events and mortality in patients with coronary artery disease.^15^, ^16^ Wang et al. revealed that elevated heart rate was independently associated with cardiac mortality among CHD patients.^17^ Lowering heart rate has been presented as an approach for improved prognosis for individuals with CHD,^16^, ^18^ whereas the role of heart rate remains to be overlooked, and heart rate control is inadequate.^15^, ^19^ Currently, research on the relationship between the specific range of heart rate and CV death in CHD patients is lacking.

The aim of this study was to assess the association between the specific range of heart rate and CV death in patients with CHD utilizing data from the Health and Nutrition Examination Survey (NHANES).

## 2 METHODS

### 2.1 Study design and population

The NHANES is a cross‐sectional survey to evaluate the health and nutritional status of the noninstitutionalized US population (https://www.cdc.gov/nchs/nhanes/index.htm). It was approved by the Institutional Review Board of the National Center for Health Statistics (NCHS), and all participants provided written informed consent. Since the open data of the NHANES are deidentified, further institutional review board approval is exempted. This retrospective cohort study collected the data of patients with CHD from the eight cycles (1999–2000, 2001–2002, 2003–2004, 2005–2006, 2007–2008, 2009–2010, 2011–2012, and 2013–2014) of the NHANES. Regarding the question “has a doctor or other health professional ever told you that you had CHD?”, persons who answered “yes” were regarded to have CHD. The pulse rate of these patients was also measured.

### 2.2 Study variables

#### 2.2.1 Outcome variable

CV death was defined in the National Death Index (NDI) as the death from diseases of heart (I00–I09, I11, I13, and I20–I51) and cerebrovascular diseases (I60–I69). Hypertensive heart disease with or without renal disease was coded as I11 and I13. Ischemic heart diseases were coded as I20–I25. Other diseases of heart were coded as I00–I09 and I26–I51 (https://www.cdc.gov/nchs/data/datalinkage/underlying_and_multiple_cause_of_death_codes.pdf). The NCHS linked mortality files (the NDI data) are person‐level files and can be linked to the public‐use NHANES data by matching on the unique person‐level SEQN (for NHANES; https://www.cdc.gov/nchs/data-linkage/mortality-public.htm). Therefore, IRB approval was not required due to the linkage to the deidentified NHANES data.

#### 2.2.2 Independent variable

Patients participated in a mobile examination center (MEC) session in the morning, afternoon, or evening. During the MEC session, trained examiners measured the patients' 30‐s pulse rate according to the standardized NHANES protocol after the patients sat quietly for 5 min. In short, the right palm of the patients faced up, and the examiners used the pads of the index and middle fingers to palpate the radial pulse on the lateral flexor surface of the wrist. A digital stopwatch or wall clock was applied to record the 30‐s pulse rate, which was then multiplied by 2 to represent the 60‐s pulse rate.^20^ Heart rate in this study was expressed by the 60‐s pulse rate. The included patients were divided into four groups based on their heart rate (beats per minute, bpm): <60, 60–69, 70–79, and ≥80 bpm.

#### 2.2.3 Covariates

Data of the included patients were obtained, including gender, age (years), race, education level, marital status, ratio of family income to poverty, hypertension, high cholesterol level, diabetes, smoking status, waist circumference (cm), body mass index (BMI, kg/m^2^), estimated glomerular filtration rate [eGFR, ml/(min*1.73 m^2^)], albumin (g/dl), blood urea nitrogen (BUN, mg/dl), heart‐related drug use, antidepressant drug use, and antihypertensive drug use.

### 2.3 Follow‐up

When the included patient died, the follow‐up was terminated. The last follow‐up time was December 31, 2015. The follow‐up time was from the MEC date to the date of death or December 31, 2015. Causes of death for these patients were recorded in death certificate records from the NDI (https://www.cdc.gov/nchs/data/datalinkage/public-use-2015-linked-mortality-file-description.pdf).

### 2.4 Statistical analysis

SAS v.9.4 (SAS Institute Inc.) was used for statistical analysis. All statistical tests were two‐sided, and the confidence level was *α* = .05. Measurement data were expressed as the mean (standard error) [Mean (SE)]. Categorical variables were described as the case number and percentage of each category [*n* (%)]. Missing data were imputed with the weighted multiple imputation method using the mice package in R v.3.6.3 (R Foundation for Statistical Computing), and five data sets were imputed (Table S1). For each imputed data set, continuous variables were imputed using means, and categorical variables were imputed using modes. Then the data before and after the imputation were compared. No significant difference was found in baseline data before and after the imputation (Table S2). Covariates were sequentially included in the single‐factor competing risk model (the Fine‐Gray model) to screen for potential confounders. These confounders were then gradually adjusted in the multi‐factor competing risk model (the Fine‐Gray model) with heart rate as the main research variable to confirm whether heart rate was an independent factor for CV death. Model 1 was a single‐factor model without adjustment, Model 2 was a multi‐factor model adjusted for common demographic data (gender, age, race, education level, and ratio of family income to poverty), and Model 3 was a multi‐factor model additionally adjusted for marital status, hypertension, high cholesterol level, diabetes, smoking status, waist circumference, BMI, eGFR, albumin, BUN, heart‐related drug use, antidepressant drug use, and antihypertensive drug use on the basis of Model 2. The competing risk category referred to non‐CV death. There is a strong and frequent association between hypertension and CHD,^21^ and hypertension acts as a risk factor for CHD.^22^, ^23^ Besides, heart rate was reported to be associated with CV death in hypertensive patients,^19^, ^24^, ^25^ and the association between heart rate and CV death in non‐hypertensive and hypertensive CHD populations was under‐investigated. Hence, we performed subgroup analysis according to whether CHD patients had hypertension, and investigated the association between heart rate and CV death in CHD patients with/without hypertension. Model 1 was a single‐factor model, Model 2 was a multi‐factor model controlled for common demographic data (gender, age, race, education level, and ratio of family income to poverty), and Model 3 was a multi‐factor model adjusted for common demographic data (gender, age, race, education level, and ratio of family income to poverty), marital status, high cholesterol level, diabetes, smoking status, waist circumference, BMI, eGFR, albumin, BUN, heart‐related drug use, antidepressant drug use, and antihypertensive drug use. Hazard ratios (HRs) and 95% confidence intervals (CIs) were calculated. *p* < .05 was deemed as statistically significant.

## 3 RESULTS

### 3.1 Patient characteristics

**Figure 1:**
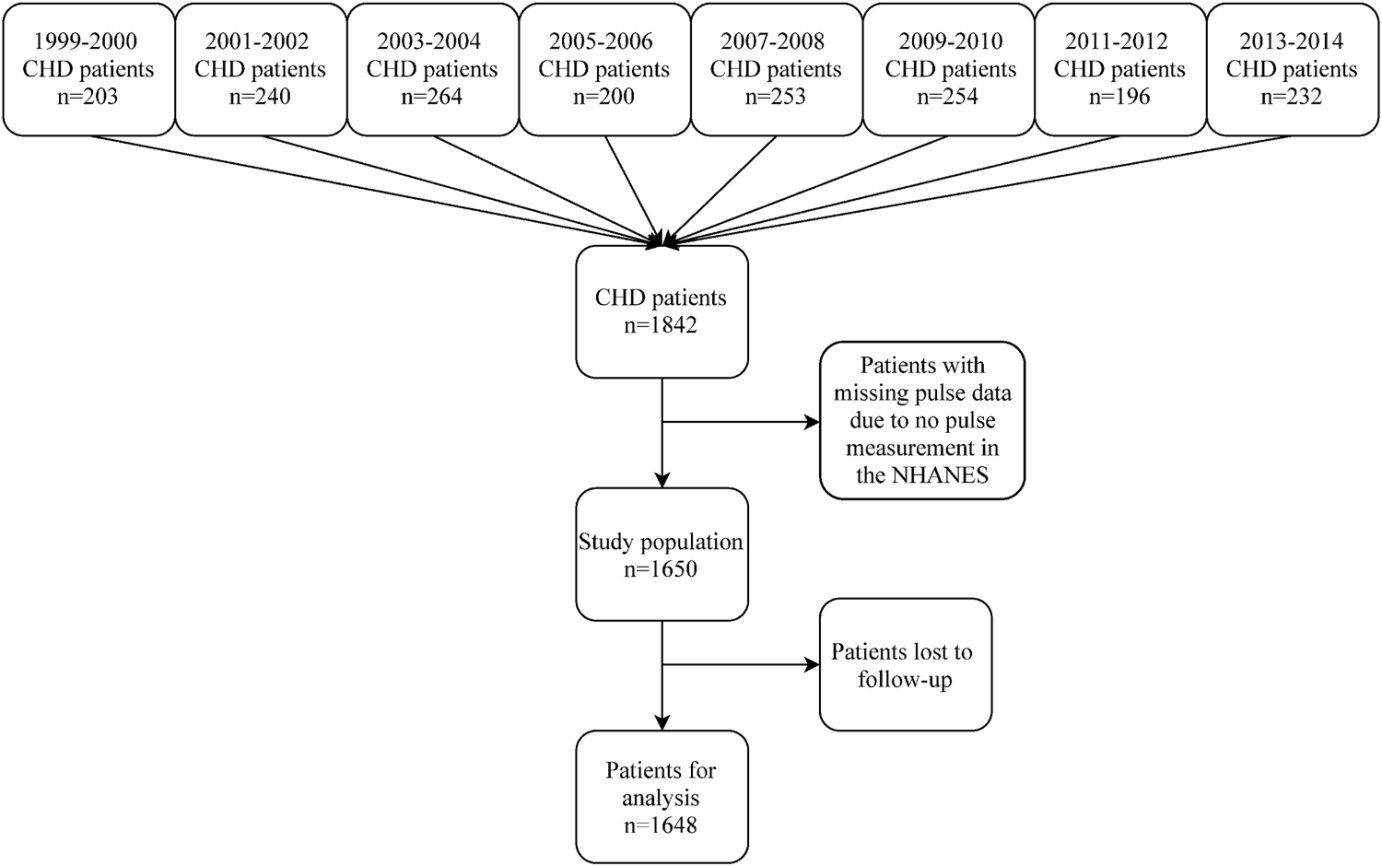
Flow chart of patient selection. CHD, coronary heart disease; NHANES, the Health and Nutrition Examination Survey

A total of 1650 patients with CHD were included, with two patients lost to follow‐up. Eventually, 1648 patients were included in this study. The flow chart of patient selection is illustrated in Figure 1. The average follow‐up time (SE) was 81.70 (2.12) months, and the longest follow‐up time was 200 months. Table 1 exhibits the basic characteristics of the study population. According to vital status, these patients were classified as alive (*n* = 986), dead from CV events (*n* = 198), and dead from non‐CV events (*n* = 464). The majority of CHD patients (65.20%) were male. The mean age and heart rate of all the patients were 66.55 years and 68.13 bpm, respectively. More patients (34.02%) had heart rate of 60–69 bpm. Most of the participants were non‐Hispanic white (82.64%) and married (63.67%). Hypertension (70.76%), high cholesterol level (73.16%), and tobacco use (64.94%) were common in these patients with CHD. The mean BMI was 29.70 kg/m^2^.

**Table 1:** Baseline characteristics of the study population

Variable	Total (*n* = 1648)	Alive (*n* = 986)	Dead from CV events (*n* = 198)	Dead from non‐CV events (*n* = 464)
**Gender, *n* (%)**				
Male	1105 (65.2)	650 (66.5)	146 (69.7)	309 (59.6)
Female	543 (34.8)	336 (33.5)	52 (30.3)	155 (40.4)
**Age (years), mean (SE)**	66.6 (0.4)	63.9 (0.4)	71.6 (0.9)	71.4 (0.8)
**Heart rate (bpm), n (%)**				
<60	354 (22.4)	223 (23.7)	48 (27.4)	83 (16.5)
60–69	572 (34.0)	344 (34.2)	61 (29.3)	167 (35.7)
70–79	408 (25.3)	243 (25.4)	47 (23.3)	118 (26.1)
≥80	314 (18.3)	176 (16.7)	42 (20.1)	96 (21.7)
**Race, *n* (%)**				
Mexican Hispanic	184 (4.0)	117 (4.0)	19 (1.8)	48 (2.8)
Non‐Hispanic white	1091 (82.6)	613 (81.5)	149 (89.2)	329 (82.7)
Non‐Hispanic black	214 (6.4)	141 (6.7)	21 (5.6)	52 (5.7)
Other	159 (7.6)	115 (7.8)	9 (3.5)	35 (8.8)
**Education level, *n* (%)**				
Less than 9th grade	311 (11.2)	148 (7.9)	42 (14.5)	121 (18.8)
9–11th grade	300 (17.8)	177 (16.1)	42 (21.0)	81 (20.8)
High school graduate/GED or equivalent	387 (25.9)	225 (24.9)	40 (23.9)	122 (29.3)
Some college or AA degree	372 (25.8)	236 (27.9)	49 (28.4)	87 (18.8)
College graduate or above	278 (19.3)	200 (23.2)	25 (12.2)	53 (12.2)
**Marital status, *n* (%)**				
Married	997 (63.7)	628 (67.2)	112 (58.6)	257 (56.5)
Not married	651 (36.3)	358 (32.8)	86 (41.4)	207 (43.5)
**Ratio of family income to poverty, mean (SE)**	2.8 (0.1)	3.0 (0.1)	2.5 (0.1)	2.4 (0.1)
**Hypertension (yes), *n* (%)**	1190 (70.8)	723 (69.9)	147 (76.2)	320 (70.5)
**High cholesterol level (yes), *n* (%)**	1168 (73.2)	726 (75.8)	135 (69.0)	307 (67.8)
**Diabetes (yes), *n* (%)**	543 (30.5)	300 (26.7)	76 (39.3)	167 (36.7)
**Smoking status (yes), *n* (%)**	1047 (64.9)	609 (63.8)	136 (70.4)	302 (65.5)
**Waist circumference (cm), mean (SE)**	105.1 (0.4)	105.7 (0.6)	106.0 (1.2)	103.2 (0.9)
**BMI (kg/m^2^), mean (SE)**	29.7 (0.2)	30.0 (0.2)	29.6 (0.5)	29.0 (0.4)
eGFR [ml/(min*1.73 m^2^)], mean (SE)	76.5 (0.6)	79.5 (0.8)	70.1 (1.6)	71.2 (1.3)
**Albumin (g/dl), mean (SE)**	4.2 (0.01)	4.2 (0.01)	4.2 (0.03)	4.2 (0.03)
**BUN (mg/dl), mean (SE)**	17.7 (0.3)	16.3 (0.3)	19.9 (1.0)	20.7 (0.6)
**Heart‐related drug use (yes), *n* (%)**	1423 (86.4)	850 (86.3)	177 (90.3)	396 (84.9)
**Antidepressant drug use (yes), *n* (%)**	175 (10.54)	98 (9.77)	21 (10.90)	56 (12.47)
**Antihypertensive drug use (yes), *n* (%)**	890 (52.11)	547 (52.46)	107 (55.03)	236 (49.82)

Abbreviations: AA, associates; BMI, body mass index; BUN, blood urea nitrogen; CHD, coronary heart disease; CV events, cardiovascular events; eGFR, estimated glomerular filtration rate; GED, General Education Development; SE, standard error.

### 3.2 Association between heart rate and CV death in all CHD patients

Gender, age, race, education level, marital status, ratio of family income to poverty, hypertension, high cholesterol level, diabetes, smoking status, waist circumference, BMI, eGFR, albumin, BUN, heart‐related drug use, antidepressant drug use, and antihypertensive drug use were illustrated to be significantly associated with the risk of CV death based on the single‐factor competing risk model (all *p* < .05, Table 2). Subsequently, the multi‐factor competing risk model was used to explore the relationship between heart rate and CV death. It was demonstrated that patients at heart rate of <60 (HR, 1.35; 95% CI, 1.34–1.36), 60–69 (HR, 1.05; 95% CI, 1.04–1.06) or ≥80 (HR, 1.39; 95% CI, 1.38–1.41) bpm had a higher risk of CV death than those at heart rate of 70–79 bpm, after adjusting for all the above covariates (Table 3, Figures S1 and S2).

**Table 2:** Factors associated with the risk of CV death in the single‐factor model

Variable	HR (95% CI)	*p*
**Gender (female)**	0.75 (0.75–0.75)	<.001
**Age**	1.05 (1.05–1.05)	<.001
**Race, *n* (%*)***		
Mexican Hispanic		
Non‐Hispanic white	2.41 (2.37–2.46)	<.001
Non‐Hispanic black	1.70 (1.67–1.74)	<.001
Other	0.92 (0.90–0.94)	<.001
**Education level**	0.93 (0.93–0.93)	<.001
**Marital status (not married)**	1.29 (1.29–1.30)	<.001
**Ratio of family income to poverty**	0.87 (0.86–0.87)	<.001
**Hypertension (no)**	0.68 (0.68–0.68)	<.001
**High cholesterol level (no)**	1.14 (1.13–1.14)	<.001
**Diabetes (no)**	0.62 (0.62–0.63)	<.001
**Smoking status (no)**	0.86 (0.86–0.87)	<.001
**Waist circumference**	1.01 (1.01–1.01)	<.001
**BMI**	1.00 (1.00–1.00)	<.001
**eGFR**	0.98 (0.98–0.98)	<.001
**Albumin**	0.66 (0.66–0.67)	<.001
**BUN**	1.02 (1.02–1.02)	<.001
**Heart‐related drug use**	1.80 (1.78–1.81)	<.001
**Antidepressant drug use**	1.10 (1.10–1.11)	<.001
**Antihypertensive drug use**	1.72 (1.70–1.73)	<.001

Abbreviations: BMI, body mass index; BUN, blood urea nitrogen; CHD, coronary heart disease; CI, confidence interval; CV death, cardiovascular death; eGFR, estimated glomerular filtration rate; HR, hazard ratio.

**Table 3:** Association between heart rate and CV death in CHD patients

Variable	Model 1		Model 2		Model 3	
	HR (95% CI)	*p*	HR (95% CI)	*p*	HR (95% CI)	
**Heart rate**						
<60	1.41 (1.40–1.42)	<.001	1.37 (1.36–1.38)	<.001	1.35 (1.34–1.36)	<.001
60–69	1.19 (1.18–1.19)	<.001	1.07 (1.07–1.08)	<.001	1.05 (1.04–1.06)	<.001
70–79	REF		REF		REF	
≥80	1.38 (1.37–1.39)	<.001	1.54 (1.53–1.55)	<.001	1.39 (1.38–1.41)	<.001
**Gender (male)**			1.59 (1.58–1.60)	<.001	1.53 (1.52–1.54)	<.001
**Age**			1.05 (1.05–1.05)	<.001	1.04 (1.04–1.04)	<.001
**Race**						
Mexican Hispanic	REF		REF		REF	
Non‐Hispanic white			1.97 (1.93–2.00)	<.001	1.74 (1.71–1.78)	<0.001
Non‐Hispanic black			1.76 (1.72–1.80)	<.001	1.52 (1.48–1.55)	<.001
Other			0.83 (0.82–0.85)	<.001	0.61 (0.60–0.62)	<.001
**Education level**			0.85 (0.85–0.86)	<.001	1.03 (1.03–1.03)	<.001
**Ratio of family income to poverty**			1.00 (1.00–1.00)	.409	0.87 (0.87–0.87)	<.001
**Marital status (married)**					0.87 (0.86–0.87)	<.001
**Hypertension (yes)**					1.40 (1.39–1.41)	<.001
**High cholesterol level (yes)**					1.03 (1.03–1.04)	<0.001
**Diabetes (yes)**					1.52 (1.51–1.52)	<.001
**Smoking status (yes)**					1.19 (1.18–1.20)	<.001
**Waist circumference**					1.02 (1.02–1.02)	
**BMI**					0.97 (0.97–0.97)	<.001
**eGFR**					0.99 (0.99–0.99)	<.001
**Albumin**					0.72 (0.71–0.72)	<.001
**BUN**					1.00 (1.00–1.00)	<.001
**Heart‐related drug use (no)**					0.87 (0.87–0.88)	<.001
**Antidepressant drug use (no)**					0.90 (0.90–0.91)	<.001
**Antihypertensive drug use (no)**					0.85 (0.84–0.85)	<.001

*Note*: Model 1, single‐factor model without adjustment.

Model 2, multi‐factor model adjusted for common demographic data (gender, age, race, education level, and ratio of family income to poverty).

Model 3, multi‐factor model adjusted for common demographic data (gender, age, race, education level, and ratio of family income to poverty), marital status, hypertension, high cholesterol level, diabetes, smoking status, waist circumference, BMI, eGFR, albumin, BUN, heart‐related drug use, antidepressant drug use, and antihypertensive drug use.

Abbreviations: BMI, body mass index; BUN, blood urea nitrogen; CI, confidence interval; CHD, coronary heart disease; CV death, cardiovascular death; eGFR, estimated glomerular filtration rate; HR, hazard ratio; REF, reference.

### 3.3 Association between heart rate and CV death in CHD patients with hypertension

Subgroup analysis revealed that compared with heart rate of 70–79 bpm, heart rate of 60–69 bpm was associated with a lower risk of CV death (HR, 0.92; 95% CI, 0.91–0.93), while heart rate of <60 (HR, 1.04; 95% CI, 1.03–1.05) or ≥80 bpm (HR, 1.22; 95% CI, 1.21–1.24) was related to a greater risk of CV death in patients with hypertension (Figure S3). In addition, for non‐hypertensive patients (Figure S4), 2.66‐, 0.61‐, 2.41‐fold elevations were shown in the risk of CV death when heart rate was <60 (HR, 3.66; 95% CI, 3.60–3.73), 60–69 (HR, 1.61; 95% CI, 1.58–1.64) and ≥80 (HR, 3.41; 95% CI, 3.35–3.48) bpm respectively, relative to 70–79 bpm (Table 4, Figure S5).

**Table 4:** Association between heart rate and CV death in CHD patients with/without hypertension

Variable	Model 1		Model 2		Model 3	
	HR (95% CI)	*p*	HR (95% CI)	*p*	HR (95% CI)	*p*
*Hypertension*						
**Heart rate**						
<60	1.41 (1.40–1.42)	<.001	1.07 (1.06–1.08)	<.001	1.04 (1.03–1.05)	.004
60–69	1.19 (1.18–1.19)	<.001	0.96 (0.96–0.97)	<.001	0.92 (0.91–0.93)	<.001
70–79	REF		REF		REF	
≥80	1.38 (1.37–1.39)	<.001	1.36 (1.34–1.37)	<.001	1.22 (1.21–1.24)	<.001
**Gender (male)**			1.70 (1.69‐1.70)	<.001	1.62 (1.60–1.63)	<.001
**Age**			1.05 (1.05‐1.05)	<.001	1.04 (1.04–1.04)	<.001
**Race**						
Mexican Hispanic	REF		REF		REF	
Non‐Hispanic white			1.82 (1.78–1.85)	<.001	1.63 (1.60–1.67)	<.001
Non‐Hispanic black			1.36 (1.32–1.39)	<.001	1.23 (1.20–1.26)	<.001
Other			0.53 (0.51–0.54)	<.001	0.39 (0.38–0.40)	<.001
**Education level**			0.81 (0.80–0.81)	<.001	1.09 (1.09–1.09)	<.001
**Ratio of family income to poverty**			1.06 (1.05–1.06)	<.001	0.83 (0.83–0.83)	<.001
**Marital status (married)**					0.83 (0.83–0.84)	<.001
**High cholesterol level (yes)**					1.03 (1.03–1.04)	<.001
**Diabetes (yes)**					1.37 (1.37–1.38)	<.001
**Smoking status (yes)**					1.13 (1.12–1.13)	<.001
**Waist circumference**					1.01 (1.01–1.01)	
**BMI**					0.98 (0.97–0.98)	<.001
**eGFR**					0.99 (0.99–0.99)	<.001
**Albumin**					0.74 (0.74–0.75)	<.001
**BUN**					1.00 (1.00–1.00)	<.001
**Heart‐related drug use (no)**					1.18 (1.17–1.19)	<.001
**Antidepressant drug use (no)**					0.82 (0.81–0.83)	<.001
**Antihypertensive drug use (no)**					0.47 (0.46–0.48)	<.001
*Non‐hypertension*						
**Heart rate**						
<60	1.41 (1.40–1.42)	<.001	2.70 (2.66–2.75)	<.001	3.66 (3.60–3.73)	<.001
60–69	1.19 (1.18–1.19)	<.001	1.24 (1.22–1.26)	<.001	1.61 (1.58–1.64)	<.001
70–79	REF		REF		REF	
≥80	1.38 (1.37–1.39)	<.001	3.84 (3.77–3.91)	<.001	3.41 (3.35–3.48)	<.001
**Gender (male)**			1.82 (1.80–1.85)	<.001	1.74 (1.72–1.77)	<.001
**Age**			1.07 (1.07–1.07)	<.001	1.06 (1.06–1.06)	<.001
**Race**						
Mexican Hispanic	REF		REF		REF	
Non‐Hispanic white			1.22 (1.18–1.27)	<.001	1.32 (1.26–1.37)	<.001
Non‐Hispanic black			2.16 (2.05–2.27)	<.001	2.31 (2.19–2.43)	<.001
Other			0.99 (0.96–1.03)	.776	0.81 (0.78–0.85)	<.001
**Education level**			0.96 (0.96–0.97)	<.001	1.04 (1.04–1.05)	<.001
**Ratio of family income to poverty**			1.01 (1.01–1.02)	<.001	1.00 (1.00–1.00)	.959
**Marital status (married)**					0.75 (0.74–0.76)	<.001
**High cholesterol level (yes)**					0.76 (0.74–0.77)	<.001
**Diabetes (yes)**					1.99 (1.97–2.02)	<.001
**Smoking status (yes)**					1.70 (1.68–1.73)	<.001
**Waist circumference**					1.00 (1.00–1.00)	
**BMI**					1.02 (1.01–1.02)	<.001
**eGFR**					0.98 (0.98–0.98)	<.001
**Albumin**					0.52 (0.51–0.53)	<.001
**BUN**					0.97 (0.97–0.98)	<.001
**Heart‐related drug use (no)**					0.96 (0.94–0.98)	<0.001
**Antidepressant drug use (no)**					0.90 (0.88–0.91)	<.001
**Antihypertensive drug use (no)**					1.41 (1.39–1.43)	<.001

*Note*: Model 1, single‐factor model without adjustment.

Model 2, multi‐factor model adjusted for common demographic data (gender, age, race, education level, and ratio of family income to poverty).

Model 3, multi‐factor model adjusted for common demographic data (gender, age, race, education level, and ratio of family income to poverty), marital status, high cholesterol level, diabetes, smoking status, waist circumference, BMI, eGFR, albumin, BUN, heart‐related drug use, antidepressant drug use, and antihypertensive drug use.

Abbreviations: BMI, body mass index; BUN, blood urea nitrogen; CHD, coronary heart disease; CI, confidence interval; CV death, cardiovascular death; eGFR, estimated glomerular filtration rate; HR, hazard ratio; REF, reference.

## 4 DISCUSSION

Heart rate has been identified as a predictor of CV risk and death,^14^ whereas the association between specific heart rate ranges and CV death is unknown for patients with CHD. The present study filled this gap via classifying 1648 CHD patients from the NHANES into <60, 60–69, 70–79, and ≥80 bpm groups, according to resting heart rate. It was found that compared with heart rate of 70–79 bpm, heart rate of <70 or ≥80 bpm was associated with a greater risk of CV death in CHD patients.

A review illustrated that high resting heart rate appeared to cause CV events mainly through ventricular arrhythmia or progressive pump failure in patients with coronary artery disease.^14^ Aune et al.^26^ conducted a meta‐analysis and found that each 10 bpm increase in heart rate raised the risks of CHD and sudden cardiac death by 7% and 9%, respectively. Greater heart rate at rest was confirmed by the Heart and Soul Study as an independent predictor for CV mortality among CHD patients.^17^ Elevation of 10 bpm in resting heart rate related to an 11% increase in CV death.^27^ Increased heart rate (80 bpm or greater) can be applied to identify an elevated risk of mortality for people with acute myocardial infarction receiving primary percutaneous coronary intervention.^28^ Further, as for patients with CHD, we demonstrated that the risk of CV death in patients with heart rate of ≥80 bpm was 1.39 times that in those with heart rate at 70–79 bpm.

The explanation of the above phenomenon might be that elevated resting heart rate is associated with higher triglyceride, total cholesterol, non‐high‐density lipoprotein cholesterol and apolipoprotein B, atherogenic lipoprotein subfractions, and endothelial dysfunction, which can facilitate CV mortality in CHD.^29^, ^30^ Resting heart rate may be a marker of underlying sympathetic nervous system activity.^31^ Over‐activity of sympathetic nerves is involved in the development of CV events.^32^, ^33^ In addition, a strong synergistic effect of inflammatory activity and concurrently increased heart rate may also relate to CV death in CHD patients.^34^

In another aspect, heart rate of <70 bpm was also presented to be associated with a greater risk of CV mortality in CHD patients. Ferrari and Fox^16^ reported the association between low heart rate and atrial fibrillation, which is related to an elevated risk of all‐cause mortality, CV mortality, and sudden cardiac death.^35^ Bradycardia can cause dispersion of atrial repolarization, which in turn triggers atrial fibrillation (the recognized mechanism of vagal‐mediated atrial fibrillation).^36^ Similarly, middle‐aged and older people in China with low baseline heart rate (<65 bpm) were shown to have a higher risk of CVD.^37^

The current cohort study specified the suitable range of heart rate associated with a reduction in the risk for CV mortality, using the NHANES data of 1648 CHD patients from 1999 to 2014. It was revealed that heart rate at 70–79 bpm was associated with a decreased risk of CV death, especially for CHD patients without hypertension. Of note, for CHD patients with hypertension, heart rate of 60–69 bpm was linked to a lower risk of CV mortality than that of 70–79 bpm. Continuous monitoring of heart rate may help to screen for health risks and offer early interventions to corresponding CHD patients.

There are some limitations in this study. First, heart rate represented by pulse rate may not be fully equal to actual heart rate, and pulse rate was only measured once. Besides, there is no data available on the validity or the intra/inter‐rater reliability of the pulse measurement. Second, variables that can lie in the causal pathway between heart rate and CV mortality, such as heart‐related drug use, may be overadjusted, which may affect the actual association between heart rate and CV mortality. Third, the findings conveyed associations, and causality cannot be determined. Fourth, our findings were based on the American population, and needs to be certified by more assessment.

In conclusion, for CHD patients, heart rate of <70 or ≥80 bpm was associated with an elevated risk of CV death, underscoring the importance of extra attention to heart rate. Properly‐designed prospective cohort studies are warranted to confirm our findings.

## CONFLICTS OF INTEREST

The authors declare no conflicts of interest.

## Supporting information

Supporting information.

Supporting information.

Supporting information.

Supporting information.

Supporting information.

Supporting information.

Supporting information.

## Data Availability

The data sets used and/or analyzed during the current study are available from the corresponding author on reasonable request.
