# Supplementary figures and images for: Association between heart rate and cardiovascular death in patients with coronary heart disease: A NHANES‐based cohort study
Source: Clin Cardiol. 2022 Mar 30;45(5):574–82. doi: 10.1002/clc.23818 (PMC9045079; doi:10.1002/clc.23818)

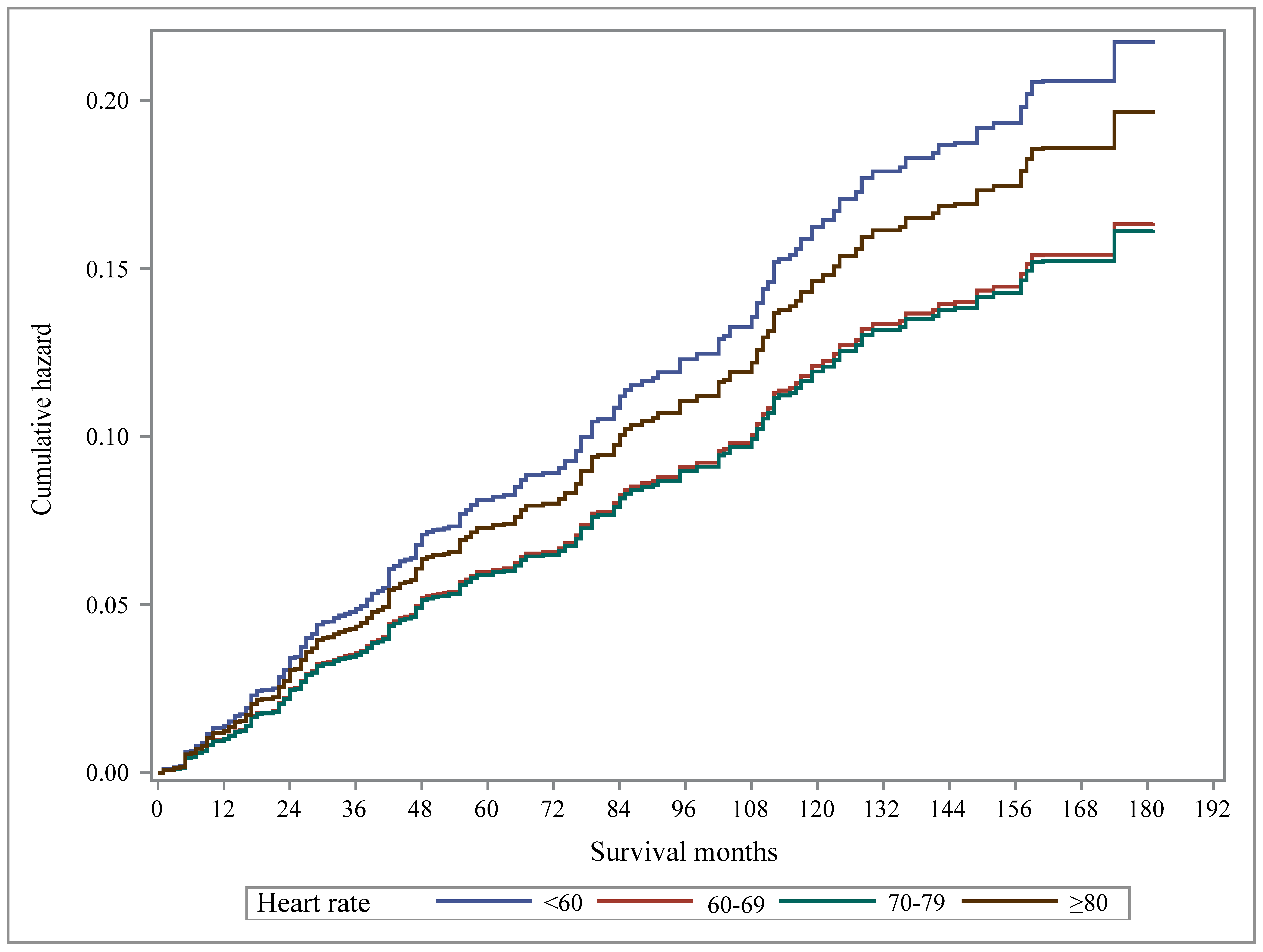

Supplement: Supplementary file 1 — Supporting information. [file CLC-45-574-s001.tif]

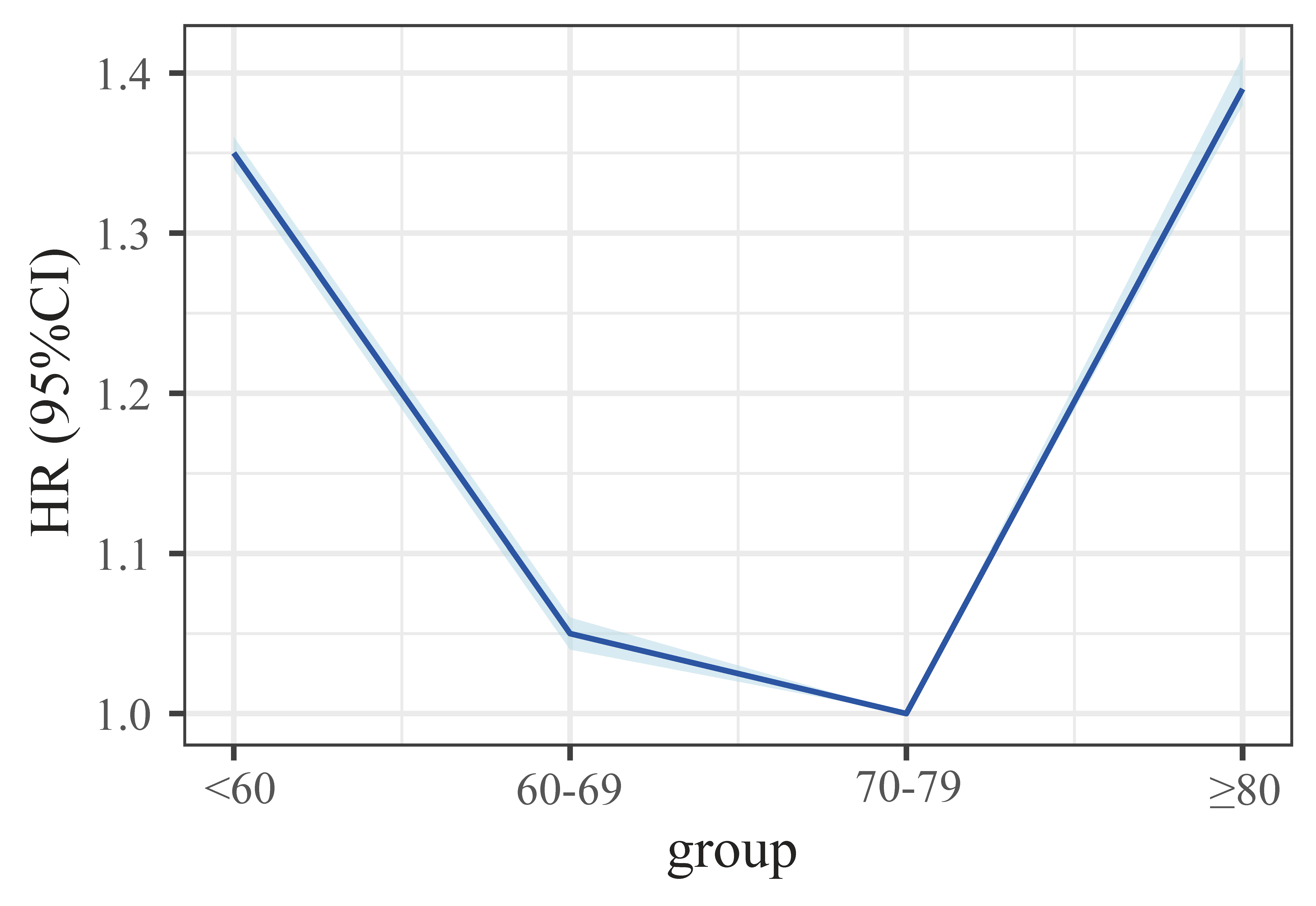

Supplement: Supplementary file 2 — Supporting information. [file CLC-45-574-s006.tif]

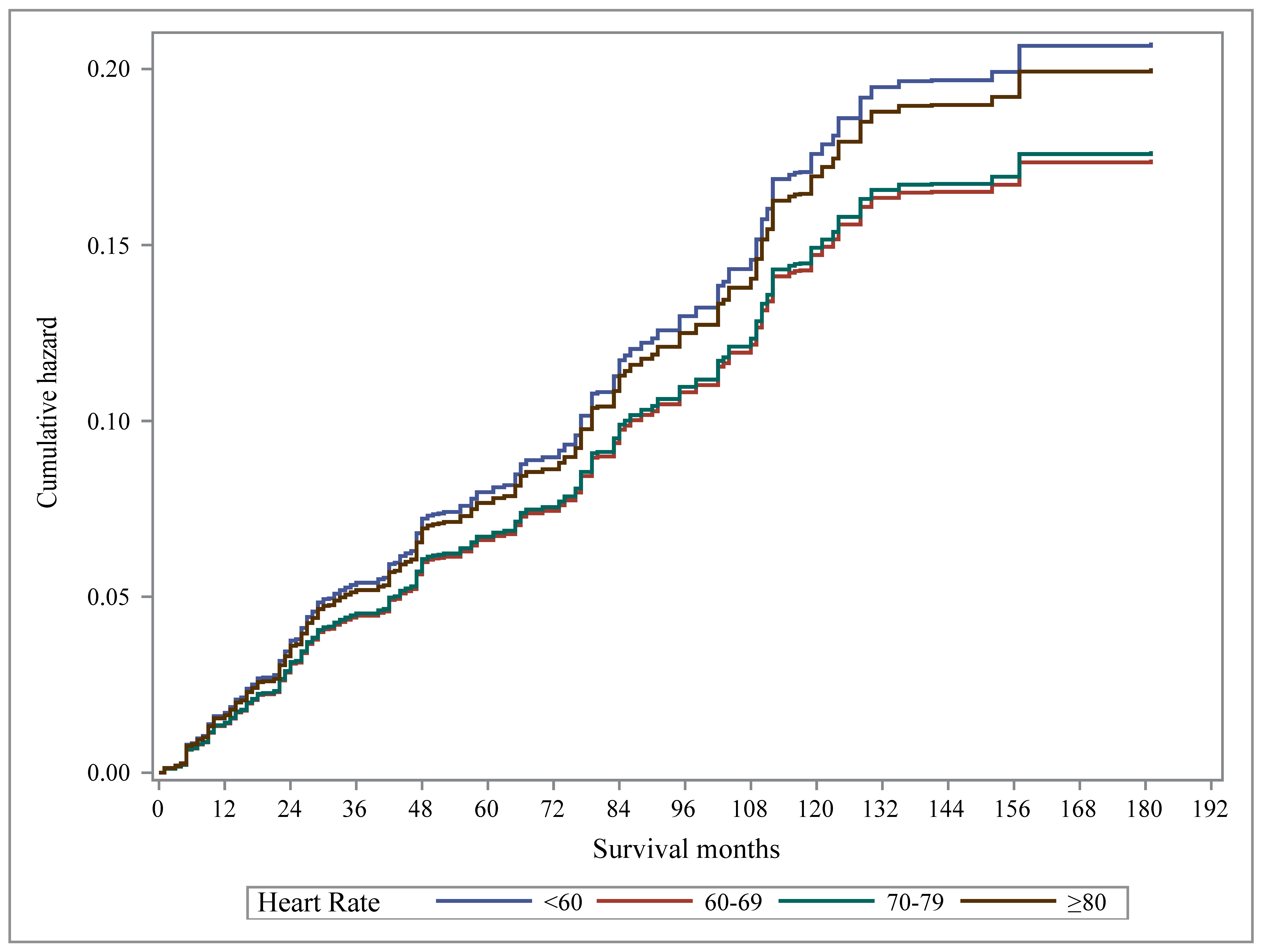

Supplement: Supplementary file 3 — Supporting information. [file CLC-45-574-s003.tif]

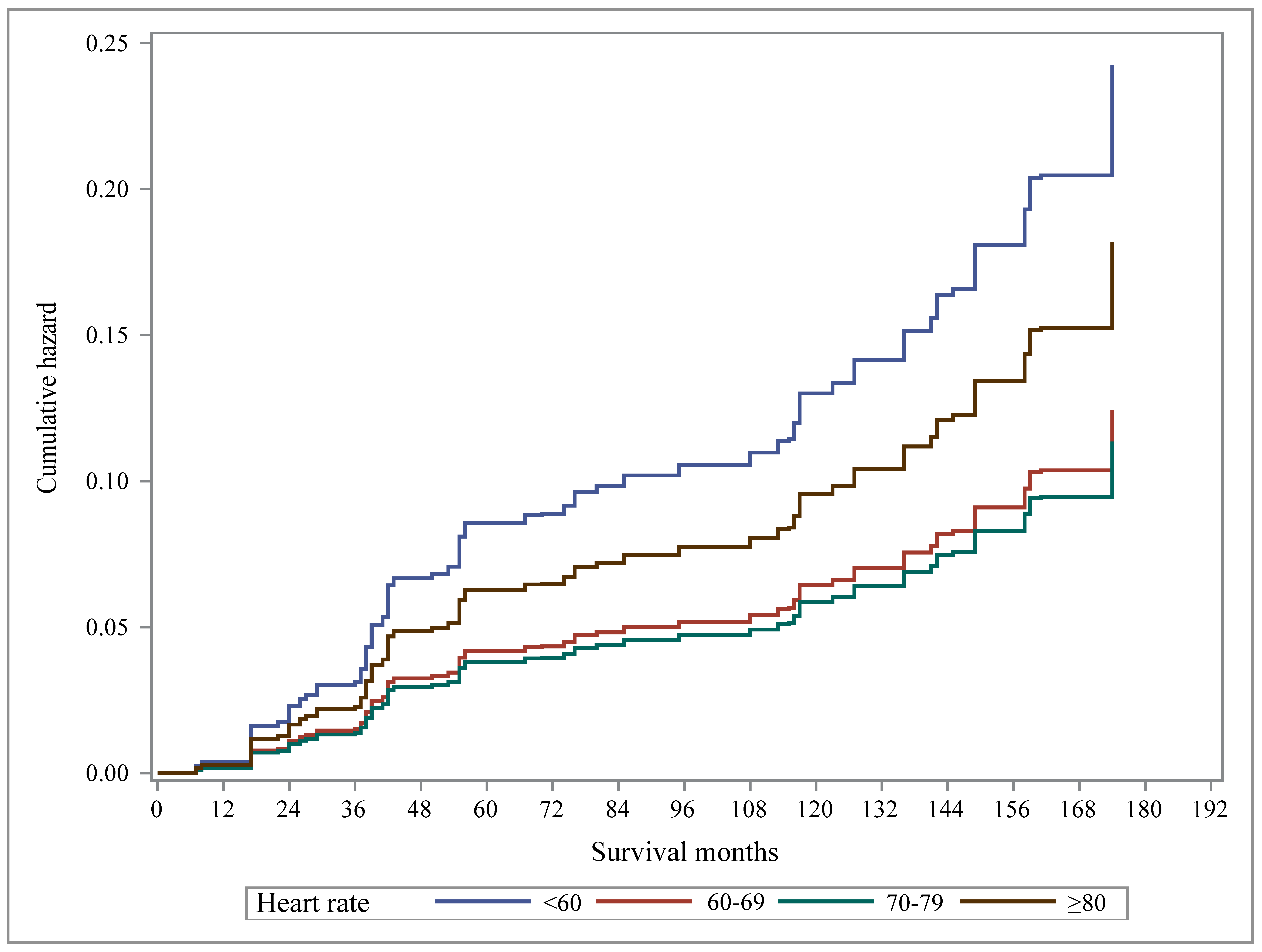

Supplement: Supplementary file 4 — Supporting information. [file CLC-45-574-s004.tif]

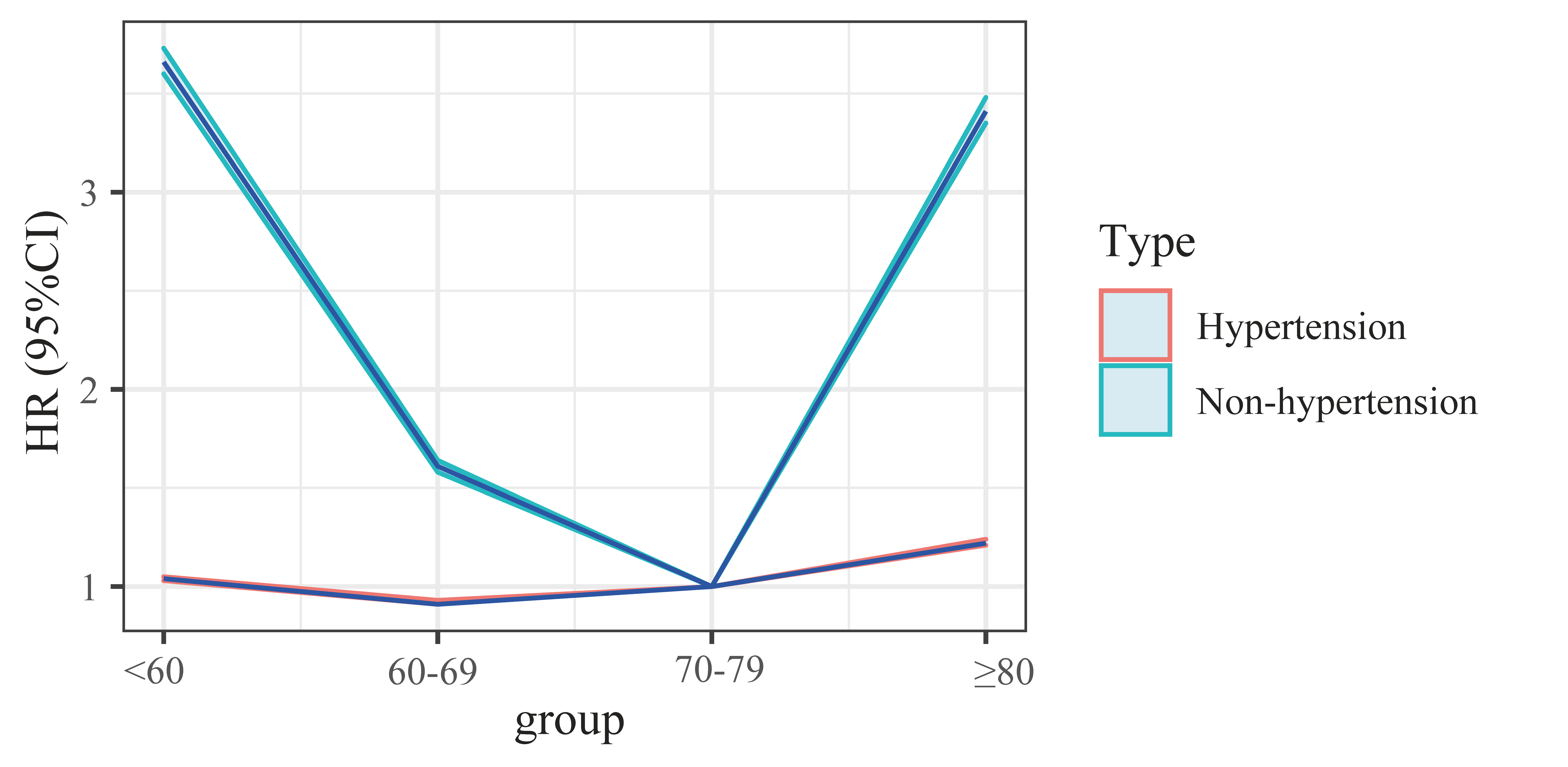

Supplement: Supplementary file 5 — Supporting information. [file CLC-45-574-s007.tif]
