# Supplementary material for: Association between heart rate and cardiovascular death in patients with coronary heart disease: A NHANES‐based cohort study
Source: Clin Cardiol. 2022 Mar 30;45(5):574–82. doi: 10.1002/clc.23818 (PMC9045079; doi:10.1002/clc.23818)
Supplement: Supplementary file 6 — Supporting information. [file CLC-45-574-s002.docx]

Supplementary Table 1 Missing data

| Variable | n (%) |
| --- | --- |
| Education level | 1 (0.1) |
| Marital status | 17 (1.0) |
| Ratio of family income to poverty | 142 (8.6) |
| Hypertension | 5 (0.3) |
| High cholesterol level | 84 (5.1) |
| Diabetes | 56 (3.4) |
| BMI | 57 (3.5) |
| Smoking status | 229 (13.9) |
| Serum creatinine | 292 (17.7) |
| Albumin | 292 (17.7) |
| BUN | 292 (17.7) |

BMI, body mass index; BUN, blood urea nitrogen.

Supplementary Table 2 Comparison of the data before and after the imputation

| Variable | Total (n=3296) | After (n=1648) | Before (n=1648) | Statistic |
| --- | --- | --- | --- | --- |
| Education Level, n (%) |  |  |  | χ^2^=3.818 |
| Less than 9th grade | 622 (11.22) | 311 (11.22) | 311 (11.22) |  |
| 9-11th grade | 600 (17.80) | 300 (17.80) | 300 (17.80) |  |
| High school graduate/GED or equivalent | 774 (25.88) | 387 (25.88) | 387 (25.89) |  |
| Some college or AA degree | 743 (25.78) | 372 (25.79) | 371 (25.77) |  |
| College graduate or above | 556 (19.32) | 278 (19.32) | 278 (19.32) |  |
| Marital status, n (%) |  |  |  | χ^2^=0.013 |
| Married | 1984 (63.65) | 997 (63.67) | 987 (63.64) |  |
| Not married | 1295 (36.35) | 651 (36.33) | 644 (36.36) |  |
| Ratio of family income to poverty, Mean (S.E.) | 2.82 (0.06) | 2.81 (0.06) | 2.83 (0.06) | t=-1.32 |
| Hypertension, n (%) |  |  |  | χ^2^=0.727 |
| Yes | 2376 (70.74) | 1190 (70.76) | 1186 (70.72) |  |
| No | 915 (29.26) | 458 (29.24) | 457 (29.28) |  |
| High cholesterol level, n (%) |  |  |  | χ^2^=1.536 |
| Yes | 2271 (73.00) | 1168 (73.16) | 1103 (72.83) |  |
| No | 941 (27.00) | 480 (26.84) | 461 (27.17) |  |
| Diabetes, n (%) |  |  |  | χ^2^=0.267 |
| Yes | 1070 (30.59) | 543 (30.52) | 527 (30.67) |  |
| No | 2170 (69.41) | 1105 (69.48) | 1065 (69.33) |  |
| Smoking status, n (%) |  |  |  | χ^2^=2.029 |
| Yes | 1933 (64.62) | 1047 (64.94) | 886 (64.25) |  |
| No | 1134 (35.38) | 601 (35.06) | 533 (35.75) |  |
| Waist circumference (cm), Mean (S.E.) | 105.09 (0.44) | 105.11 (0.44) | 105.07 (0.43) | t=0.31 |
| BMI (kg/m^2^), Mean (S.E.) | 29.69 (0.19) | 29.70 (0.20) | 29.67 (0.19) | t=0.67 |
| Serum creatinine, Mean (S.E.) | 1.09 (0.01) | 1.09 (0.01) | 1.09 (0.01) | t=1.24 |
| Albumin, Mean (S.E.) | 4.20 (0.01) | 4.20 (0.01) | 4.20 (0.01) | t=-0.60 |
| BUN, Mean (S.E.) | 17.73 (0.28) | 17.74 (0.26) | 17.71 (0.30) | t=0.29 |

GED, General Education Development; AA, associates; S.E., standard error; BMI, body mass index; BUN, blood urea nitrogen.
