# Supplementary material for: Association between heart rate and cardiovascular death in patients with coronary heart disease: A NHANES‐based cohort study
Source: Clin Cardiol. 2022 Mar 30;45(5):574–82. doi: 10.1002/clc.23818 (PMC9045079; doi:10.1002/clc.23818)
Supplement: Supplementary file 7 — Supporting information. [file CLC-45-574-s005.docx]

Supplementary Figure 1 Cumulative hazard of CV death in CHD patients of different heart rate. CHD, coronary heart disease; CV death, cardiovascular death.

Supplementary Figure 2 Association between heart rate and CV death in all CHD patients. CHD, coronary heart disease; CV death, cardiovascular death; HR, Hazard ratios; CI, confidence interval.

Supplementary Figure 3 Cumulative hazard of CV death in CHD patients with hypertension of different heart rate. CHD, coronary heart disease; CV death, cardiovascular death.

Supplementary Figure 4 Cumulative hazard of CV death in CHD patients without hypertension of different heart rate. CHD, coronary heart disease; CV death, cardiovascular death.

Supplementary Figure 5 Association between heart rate and CV death in CHD patients with hypertension. CHD, coronary heart disease; CV death, cardiovascular death; HR, Hazard ratios; CI, confidence interval.
